# Roles of RNA Methylation on Tumor Immunity and Clinical Implications

**DOI:** 10.3389/fimmu.2021.641507

**Published:** 2021-03-10

**Authors:** Maorun Zhang, Junmin Song, Weitang Yuan, Wei Zhang, Zhenqiang Sun

**Affiliations:** ^1^Department of Colorectal Surgery, The First Affiliated Hospital of Zhengzhou University, Zhengzhou, China; ^2^Academy of Medical Sciences, Zhengzhou University, Zhengzhou, China

**Keywords:** RNA methylation, M6A, immunoregulation, tumor immunity, immunotherapy

## Abstract

RNA methylation is a kind of RNA modification that exists widely in eukaryotes and prokaryotes. RNA methylation occurs not only in mRNA but also in ncRNA. According to the different sites of methylation, RNA methylation includes m^6^A, m^5^C, m^7^G, and 2-O-methylation modifications. Modifications affect the splicing, nucleation, stability and immunogenicity of RNA. RNA methylation is involved in many physiological and pathological processes. In the immune system, especially for tumor immunity, RNA methylation affects the maturation and response function of immune cells. Through the influence of RNA immunogenicity and innate immune components, modifications regulate the innate immunity of the body. Some recent studies verified that RNA methylation can regulate tumor immunity, which also provides a new idea for the future of treating immunological diseases and tumor immunotherapy.

## Introduction

More than 160 chemical modifications have been found in RNA (1, 2). Of these modifications, RNA methylation is a post-transcriptional modification that exists widely in eukaryotes and prokaryotes (3, 4). Methylation is the transfer of a methyl group from an active methyl compound, to another compound. As a post-transcriptional modification, RNA methylation regulates RNA splicing (5, 6), nuclear export (7), stability (8–10), translation (11–13), DNA damage repair (14), initiation of miRNA biogenesis (15) and immunogenicity (16) and then affects cellular differentiation (17), embryonic development (18), spermatogenesis (19), sex determination (20), learning and memory (12), immune response (21) and the occurrence and development of cancer (22–24). Since its discovery in the 1970s, m^6^A methylation has been considered to be the most abundant methylation modification in the mRNA of eukaryotes (25). With the discovery of methylases and demethylases, the view that m^6^A methylation is dynamic and reversible has also been confirmed (26, 27). The discovery of recognition factors provides a deeper understanding of RNA methylation (7, 8, 13).

In recent years, RNA methylation has been proven to be related to a variety of human physiologies and diseases, especially tumor immunity (24, 28–30). RNA methylation plays an important role in regulating normal physiological processes, and abnormal methylation will lead to diseases and cancers (28, 31–33). This provides new insights for us to understand diseases from the perspective of RNA methylation and find new treatment methods. In this review, we will focus on the relationship between RNA methylation and tumor immunity. When methylation acts on different RNAs, it will directly or indirectly affect immunity especially tumor immunity (21, 34), which means that RNA methylation has the potential to be applied to the treatment of a variety of diseases in the future, including autoimmune diseases and cancers.

## Biological Features of RNA Methylation

### Types of RNA Methylation

#### m^6^A

N6-methyladenosine (m^6^A) is generated by the methylation of adenosine at the N6 position. M^6^A methylation was discovered in the 1970s and is the most abundant modification in mammalian mRNA (25). In addition to being abundant in mammals, m^6^A modification is enriched in eukaryotes such as plants (35) and yeasts (36). In addition, m^6^A modification was also found in prokaryotes such as bacteria (37). M^6^A is enriched at exons, stop codons and 3′ UTRs of RNA and mainly on adenine in the RRACH sequence, where R is guanine or adenine and H is uracil, adenine or cytosine (38). M^6^A modification is a reversible process. Methyltransferase complexes, including METTL3, METTL14 and WTAP, methylate RNA (39). Demethylases, such as FTO and ALKBH5, demethylate RNA (27, 40). M^6^A binding proteins, such as YTHDC1/2 and YTHDF1/2/3, recognize and bind methylated RNA to perform functions (5, 8, 12, 13, 19). M^6^A regulates the processing (15), translation (11–13) and stability of RNA (9, 10) and controls a variety of biological functions, including spermatogenesis (19) and stem cell differentiation (17).

#### m^5^C

5-Methylcytidine (m^5^C) is a methylation modification of the fifth N of cytosine. M^5^C methylation is found in a variety of RNAs, including tRNAs (41), rRNAs (42), mRNAs (43), and sRNAs (42). M^5^C methylation affects the structural and metabolic stability of tRNAs (41) and plays an important role in the translation of tRNAs (44). M^5^C modification of mRNAs regulates their export (45). Common m^5^C methylases include DNMT2 (46) and Nsun2 (47). M^5^C can regulate cell division (48) and protein synthesis (49).

#### m^7^G

N7-methylguanosine (m^7^G) is a modification of the seventh N of RNA guanine with a methyl group. M^7^G modification improves the stability of mRNA (50, 51). In addition, m^7^G regulates cell differentiation (52). In addition to being present on mRNAs, m^7^G modification also exists on tRNAs, rRNAs and miRNAs (53–55). M^7^G methylation complexes include METTL1 and WDR4 (52).

#### 2-O-Methylation

2-O-Methylation occurs in the methylation of RNA2′-OH and is widely found on tRNAs, rRNAs, and mRNAs in mammalian cells (1). 2-O-methylation regulates the splicing of pre-mRNAs and stability of small RNAs (56, 57).

### RNA Types With Methylation Modification

RNA methylation exists in all types of RNAs, including mRNAs, tRNAs, rRNAs and some other ncRNAs.

#### mRNA

Methylation modifications in mRNA include m^6^A, m^5^C, and m^7^G. M^6^A methylation is the most common in mRNA. Methylation modification regulates mRNA transcription, nucleation, stability and translation and thus controls the expression of proteins (6, 11, 12, 40). Methylation in mRNA also plays a role in immune regulation. For example, N6-methyladenosine modification of an mRNA encoding a lysosomal protease increases its translation, which inhibits the presentation of tumor antigens (58).

#### Non-coding RNA

In addition to mRNAs that encode proteins being methylated, some ncRNAs can also be methylated. It has been shown that RNA methylation modification plays a role in the initiation of miRNA biogenesis (15). After being methylated, some immunogenic ncRNAs lose their immunogenicity and no longer have the ability to activate the immune response. N6-methyladenosine-modified self-circRNA suppresses circRNA immunity (59). A study confirmed that m^6^A methylation of lnc-Dpf3 increases CCR7-mediated DC migration (34).

### Regulatory Mechanism of RNA Methylation

RNA methylation is a dynamic and reversible biological process that is regulated by methyltransferases and demethylases. The methylated RNA is recognized and bound by specific binding proteins and plays a corresponding role. Methyltransferases, demethylases and binding proteins are known as writers, erasers and readers, respectively.

#### Writers

RNA methylation is catalyzed by RNA methyltransferase complexes called “writers.” METTL3 was the first component identified in such a complex. Then, WTAP and METTL14 were confirmed to be components of RNA methyltransferase complexes. METTL14 reacts with METTL3 at a ratio of 1:1 to form a dimer. WTAP does not have the ability to catalyze methylation. It acts as a scaffold in the methylase complex and regulates the recruitment of the m^6^A methyltransferase complex to mRNA targets (39, 60, 61). KIAA1429, RNA binding motif protein 15/15B (RBM15/15B), ZC3H13, ZCCHC4 and METTL16 are also involved in mediating the methylation of RNA (62–66).

#### Erasers

Methylated RNA is demethylated by demethylases called “erasers,” which make reversible methylation of RNA possible. The main demethylases include FTO and ALKBH5, which have been shown to affect mRNA export and RNA metabolism (27, 40).

#### Readers

Methylated RNA binds to specific RNA-binding proteins to perform corresponding functions. These binding proteins are called “readers” and mainly include the YTH family members YTHDC1/2 and YTHDF1/2/3 and insulin-like growth factor 2 mRNA binding proteins (IGF2BP1/2/3). YTHDF1 binds to the translation initiator and ribosome to promote the translation of mRNA (13). YTHDF2 selectively recognizes and regulates the degradation of mRNA (8, 9). YTHDF3 cooperates with YTHDF1 and YTHDF2 to accelerate the translation and degradation of mRNA, respectively (67, 68). Mainly located in the nucleus, YTHDC1 recruits splicing factors and regulates the shearing and nucleation of mRNA (5, 7, 69). YTHDC2, as a methylation-binding protein, promotes translation and regulates spermatogenesis (19, 70). IGF2BPs, as methylation-binding proteins, regulate the stability and translation of RNA (71).

## Different Immunoregulation Types Mediated by RNA Methylation

### T Cells

T cells play an important role in immune regulation. T cells participate in adaptive immunity and secrete cytokines. The imbalance of T cell homeostasis is associated with some autoimmune diseases (72). T cells are also involved in the process of tumor immunity (73).

**Figure 1:**
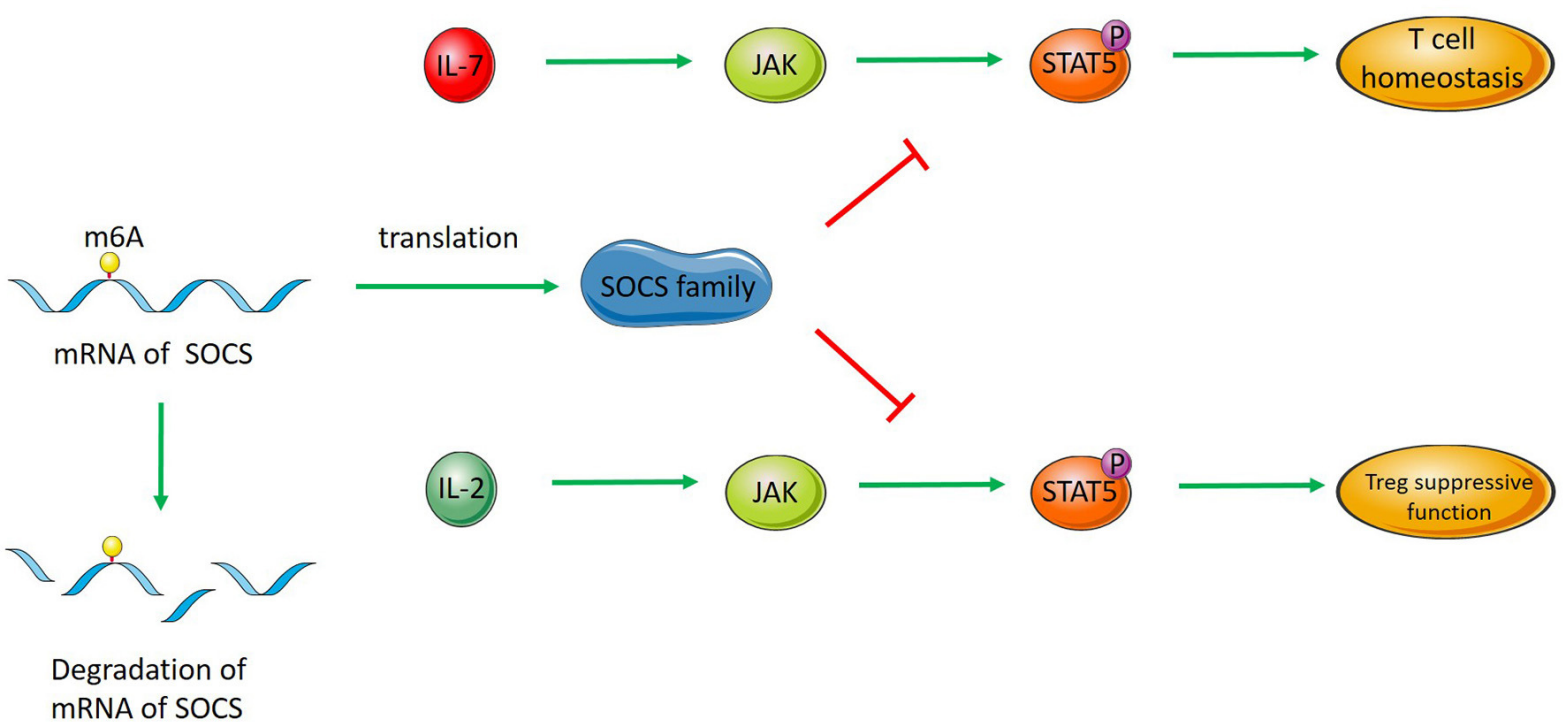
RNA methylation regulates T cells and Tregs. SOCS RNA modified by m^6^A methylation is degraded, which decreases the level of SOCS family members. SOCS proteins inhibit the activation of STAT5 in the IL-7/STAT5 pathway and regulate T cell homeostasis. SOCS proteins inhibit the activation of STAT5 in the IL-2/STAT5 pathway, thus regulating the inhibitory function of Tregs.

One study proved that RNA methylation played an essential role in maintaining T cell homeostasis. The absence of METTL3 makes T cells stay in the naive T cell stage for a longer time via METTL3-mediated m^6^A methylation targeting the IL-7/STAT5/SOCS pathway (Figure 1) (21). In addition to maintaining T cell homeostasis and differentiation, RNA methylation also promotes the expression of immune factors in T cells. IL-17 participates in the immune response and is related to some immune diseases. Nsun2 mediated the methylation of IL-17 mRNA in T cells, promoted the translation of IL-17 mRNA and increased the expression of IL-17 (74). Furthermore, in tumor immunity, the initiation of the immune response of T cells in YTHDF1-deficient mice is enhanced, featuring a stronger antitumour CD8+ T cell response (58).

Follicular helper T cell (Tfh) is a unique CD4+T cell, which can promote the response of B cells. Inducible costimulator (ICOS) plays an important role in the initiation and development of Tfh cells. A recent study found that Von Hippel–Lindau (VHL) promotes the initiation of Tfh cells through glycolysis mediated by HIF-1 α. In this process, HIF-1 α induced GAPDH to promote ICOS mRNA degradation through m6A modification, thus regulating the expression of ICOS. Mechanistically, enhanced glycolysis induced the gene expression of METTL3 and Mettl14 methyltransferase complex, and catalyzed the m6A modification of ICOS mRNA, thus inhibiting the expression of ICOS (75).

### DCs

Dendritic cells (DCs) are antigen-presenting cells that ingest, process and present antigens. Immature DCs have strong migration ability, and mature DCs stimulate and activate T cells. DCs regulate T cell differentiation and play an important role in both innate immunity and adaptive immunity. Abnormalities in the quantity and function of DCs cause colitis, among other diseases (76–79).

Methylation of mRNA affects protein expression in dendritic cells (80). N6-methyladenosine modification mediated by the methyltransferase METTL3 promotes the activation of DCs and enhances the ability of DCs to stimulate T cell activation (81). Demethylation of Lnc-Dpf3 mRNA increases the level of Lnc-Dpf3, which in turn enhances the inhibition of Lnc-Dpf3 on DC migration (34). DCs are stimulated after being exposed to non-self components. RNA transcribed *in vitro*, such as in mammalian necrotic cells, stimulates DC activation (82). RNAs with methylation modifications, including m^6^A, m^5^C, and 2-O- methylation, attenuate or eliminate this stimulation. The higher the level of modification, the stronger the inhibition of stimulation (16).

In tumor immunity, deletion of YTHDF1 enhances the tumor antigen cross-presentation ability of DCs and promotes the activation of T cells. In mice, an RNA encoding a lysosomal protease can be methylated. The methylated RNA interacts with YTHDF1, which promotes the translation of RNA. In YTHDF1^−/−^ mice, the translation of methylated RNA is not promoted, causing a low level of lysosomal protein. Limiting the degradation ability of lysosomal proteases can prevent the destruction of tumor antigens, which enhances the ability of DCs to present tumor antigens and promotes the activation of T cells (58).

### Tregs

Regulatory T cells (Tregs) play an important role in maintaining immunological self-tolerance and autoimmunity (83, 84). N6-methyladenosine modification has been shown to maintain the inhibitory function of Tregs. Tong reported that mice with m^6^A-deficient Tregs showed severe autoimmune diseases and died 8–9 weeks later. Naive T cells cocultured with Tregs without m^6^A modification showed increased proliferation, which indicates that Tregs without m^6^A modification lose their ability to inhibit T cell proliferation. In Tregs with METTL3 defects, SOCS family mRNAs reduce m^6^A methylation and increase stability, which increases the level of SOCS protein. The SOCS protein regulates the inhibitory function of Tregs by the IL2-STAT5 signaling pathway. Previous studies have found that the IL2-STAT5 pathway can regulate the function of Tregs. Methylation modifications regulate the function of Tregs by affecting those RNAs that encode inhibitor proteins of this pathway (Figure 1) (85).

### Innate Immunity

In innate immunity, macrophages are involved in regulating tissue dynamic balance, defending against viral infection and inflammation. Under different conditions, it can be polarized to M1 and M2. M1 has anti-tumor activity. M2 macrophages can inhibit inflammation, promote angiogenesis and tissue repair, and also participate in tumor metastasis (86–89).

A recent study found that silent FTO can inhibit the polarization of M1 and M2. NF-κB signal pathway is the main activation pathway of M1 polarization. It has been found that knockout of FTO can inhibit the phosphorylation of NF- κ B signal pathway to inhibit M1 polarization. However, it has not been proved whether RNA methylation is involved in this process. In addition, STAT1 promotes M1 polarization, while PPAR- γ promotes M2 polarization. Knockout of FTO reduces the stability of STAT1 and PPAR- γ mRNA through YTHDF2-mediated degradation, which results in the inhibition of polarization of M1 and M2 (90). In an earlier study, METTL3 promoted the methylation of STAT1 and enhanced the stability of STAT1mRNA, up-regulated the expression of STAT1 and promoted the polarization of M1. However, in this process, the reader that recognizes the methylation and enhances the stability of mRNA has not been found (91). RNA-binding motif 4 (RBM4) interacts with YTHDF2 to degrade M6A modified STAT1mRNA to regulate glycolysis and M1 macrophage polarization (92). In a recent study on acute coronary syndrome, it was found that IRF-1 promoted the expression of methyltransferase METTL3 in macrophages and inhibited expression of circ_0029589 by regulating its m^6^A modification, thus promoting the pyroptosis of macrophages (93).

In innate immunity, the immune system initiates a series of immune responses by recognizing foreign components. These foreign components are recognized by receptors located in the cell membrane or cytoplasm, such as Toll-like receptors and RIG-I-like receptors. After the receptor recognizes the foreign component, it stimulates the immune response and produces cytokines.

Toll-like receptors (TLRs) recognize foreign components and induce the immune response. Foreign RNAs recognized by TLRs stimulate the immune system and trigger immune responses with the production of type I interferon (82, 94). RNAs containing nucleotide modifications, including RNA methylation, suppress or avoid the activation of innate immunity (95, 96). Previous research has proven that methylation modifications inhibit the ability of RNAs to stimulate TLR3 and block the stimulation of TLR7 and TLR8 (16). Similarly, protein kinase R (PKR) is an innate immune sensor that recognizes foreign RNA and initiates innate immunity. Retinoic acid-inducible gene I (RIG-I) is a cytoplasmic innate immune receptor that recognizes RNAs of invasive pathogens (97). RNA modification, including methylation, eliminates or suppresses stimulation of PKR and RIG (98, 99).

As non-coding RNAs, foreign circRNAs have immunogenicity and act as adjuvants to induce T cell activation and antibody production (100). M^6^A modification can significantly inhibit the immune activation of circRNAs. RIG-I binds to foreign circRNAs, and with the participation of K63-polyubiquitin, it stimulates mitochondrial antiviral signaling protein (MAVS) filamentation and ultimately stimulates IRF-3 dimerization. Foreign circRNAs modified by m^6^A cannot stimulate Mavs filamentation and IRF-3 dimerization after being bound by RIG-I, and YTHDF2 binding to methylated circRNA inhibits circRNA immunity. *In vivo*, the immunogenicity of circRNAs as adjuvants is also inhibited by m^6^A modification (59).

In addition to affecting the immunogenicity of RNA itself, methylation modification also affects innate immunity by regulating innate immune components. Type I interferon is key for antiviral immunity (101). M^6^A regulates the production of type I interferon, which is stimulated by viruses. The deletion of METTL14 increases the level of type I interferon mRNA, and the deletion of ALKBH5 decreases the level of type I interferon mRNA, which regulates the level of type I interferon. Without the m^6^A modification, the type I interferon mRNA is more stable, which means a stronger immune response (102).

In vesicular stomatitis virus infection, knockout of the eraser ALKBH5 increases the level of interferon induced by the virus, while overexpression of ALKBH5 decreases the level of interferon. ALKBH5, as a demethylase, demethylates the transcripts of some antiviral proteins, such as Mavs, Traf3 and Traf6. These demethylated transcripts remain in the nucleus, and translation is inhibited, thus inhibiting the production of interferon (103).

**Figure 2:**
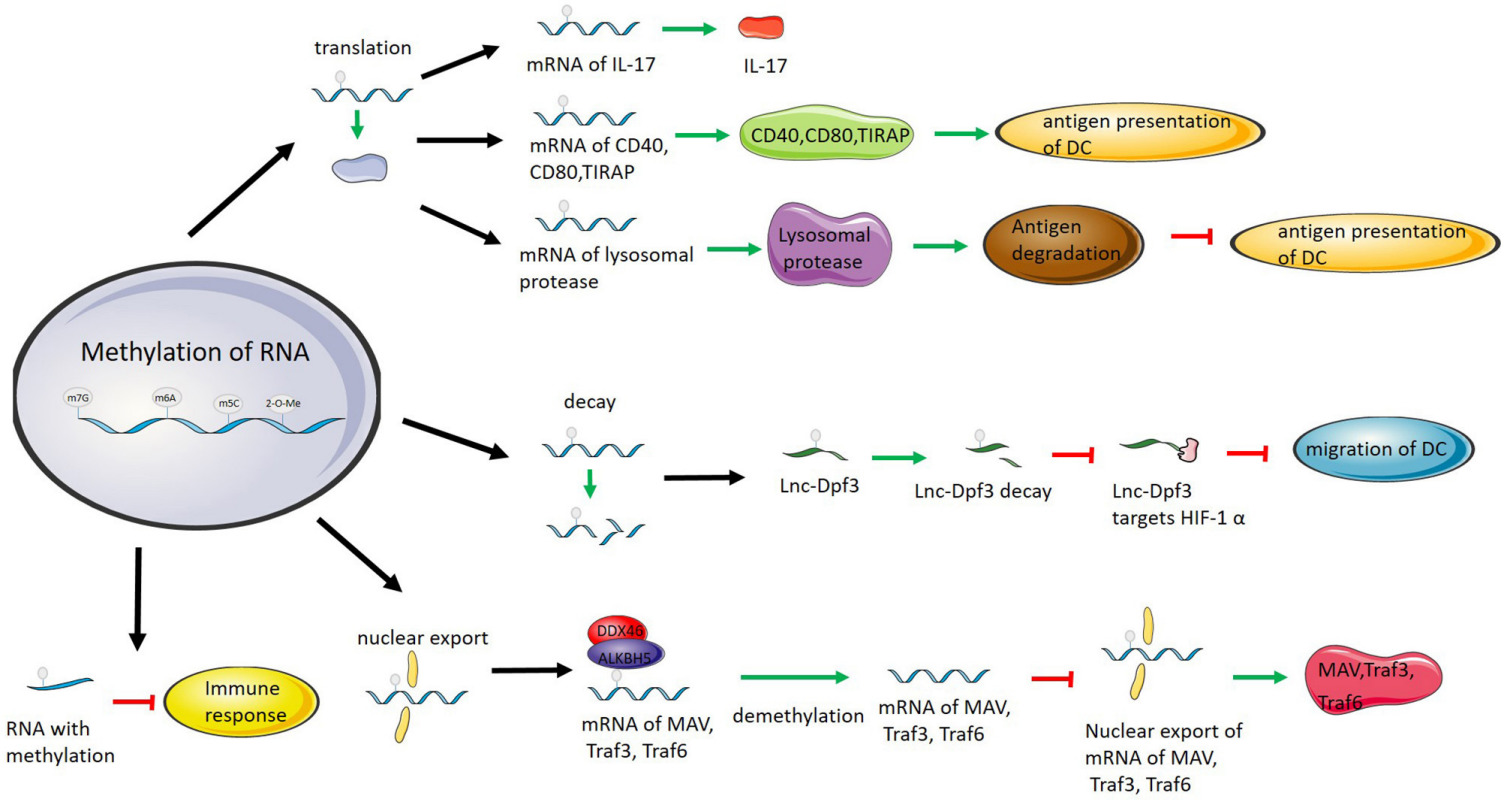
RNA methylation regulates immunity by affecting RNA translation, degradation, and nuclear export and directly inhibiting RNA immunogenicity. RNA methylation promotes IL-17 translation. RNA methylation promotes the translation of CD40, CD80, and TIRAP and improves antigen presentation by DCs. RNA methylation promotes the translation of lysosomal proteases, degrades antigens and inhibits antigen presentation by DCs. RNA methylation promotes Lnc-Dpf3 degradation to prevent Lnc-Dpf3 from inhibiting DC migration by targeting HIF-1α. DDX46 recruits ALKBH5 to demethylate MAV, Traf3, and Traf6 mRNAs, inhibit the enucleation of mRNAs, and thus inhibit the immune function of MAV, Traf3, and Traf6. Methylation of RNAs suppresses the immunogenicity of RNAs and eliminates or weakens the ability of RNAs to activate the immune response.

Methylation modification can regulate innate immunity not only by affecting the molecules involved in the immune response but also by directly affecting antigens. However, the function of methylation is different between the two mechanisms. The former mainly affects the protein translated by RNA, while the latter directly affects the immunogenicity of RNAs (Figure 2).

## Various Immunoregulation Mechanism of RNA Methylation

RNA methylation regulates immunity through a variety of mechanisms.

The transcripts encoding lysosomal proteases are recognized by YTHDF1 after methylation, and YTHDF1 combines with the methylated transcripts to promote the translation of the transcripts and increase the level of lysosomal proteases. More lysosomal proteases degrade phagocytosed tumor antigens, and DCs inhibit the presentation of antigens, resulting in immune escape (58).

In DCs, the transcripts of the immune components CD40, CD80, and Tirap are methylated by METTL3. Methylated CD40 and CD80 mRNAs are recognized by YTHDF1, and the translation of CD40, CD80 and Tirap is enhanced. When DCs express more CD40, CD80, and Tirap, the antigen presentation function of DCs is enhanced (81). Methylation of lnc-Dpf3 affects DC migration. The methylated lnc-Dpf3 is recognized and bound by YTHDF2, which enhances the degradation of lnc-Dpf3. CCR7 stimulation decreases the methylation level of lnc-Dpf3. When the methylation level of Lnc-Dpf3 decreases, the level of Lnc-Dpf3 increases due to decreased degradation. Rapid glycolytic reprogramming is triggered when DCs are stimulated to meet metabolic requirements. Increased Lnc-Dpf3 targets HIF-1α and inhibits HIF-1α-dependent transcription of the glycolysis gene LDHA. Glycolysis and migration of DCs are inhibited (34).

M^6^A methylation regulates the proliferation and differentiation of immature T cells by affecting the degradation of SOCS family mRNAs in the IL7/STAT5/SOCS pathway. In METTL3-deficient T cells, the degradation of SOCS family member mRNA transcription is decreased. The expression of STAT5 inhibitory protein is increased, which blocks the stimulation of T cell proliferation and differentiation by IL-7 and makes T cells exist for a longer time in the immature T cell stage (21).

Similar to its regulation of T cell proliferation and differentiation, m^6^A regulates the inhibitory function of Tregs through the IL2-STAT5 signaling pathway. In Tregs with METTL3 conditional knockout, the mRNA stability of SOCS family members with decreased m^6^A methylation increased, and the level of SOCS protein increased. The increased SOCS proteins inhibited the phosphorylation of STAT5 stimulated by IL-2, which in turn affected the inhibitory function of Tregs (85).

DDX46 belongs to the DEAD-box family. Most of the family members are located in the nucleus and participate in the metabolism of RNA, which plays an important role in antiviral immunity. DDX46 is located in the nucleus and recruits ALKBH5 in antiviral immunity. ALKBH5, as an eraser, can demethylate the mRNA of antiviral substances such as Mavs, Traf3, and Traf6. After demethylation, the nucleation of mRNA is inhibited, translation of mRNA is blocked, and the expression of antiviral molecules is reduced. Knockout of DDX46 *in vivo* can significantly improve the antiviral response of the body (103).

In terms of effects on immunity, on the one hand, RNA methylation affects the stability, translation and nuclear export of RNAs after modification and thus affects the levels of RNAs and proteins, which are involved in the immune process. In addition, it also affects immunity by directly affecting the immunogenicity of RNA and reduces the immune stimulation by RNA.

## Immunoregulation of RNA Methylation in Various Cancers

**Figure 3:**
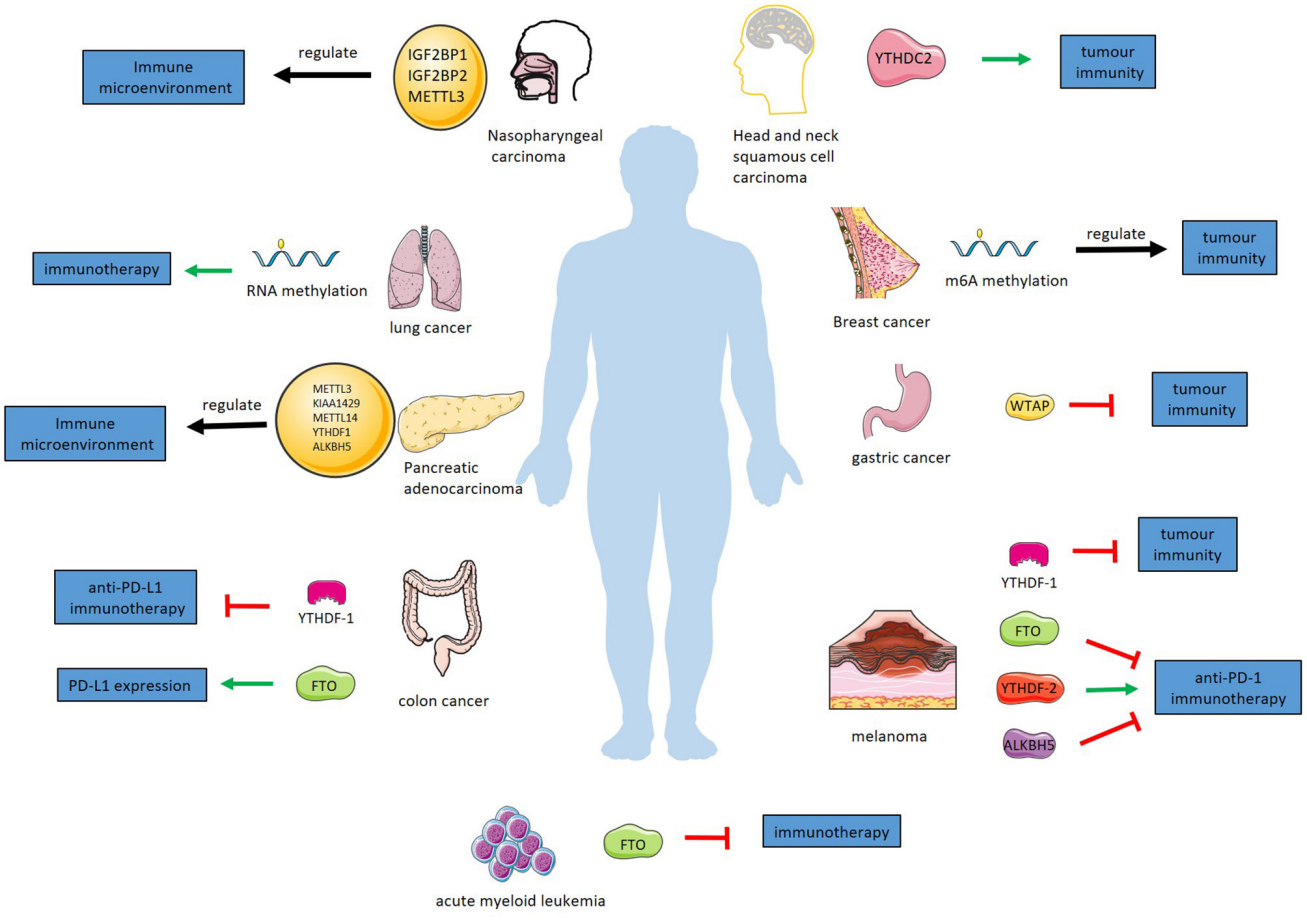
RNA methylation regulates tumor immunity in various cancers. In melanoma, YTHDF-1 inhibits tumor immunity. In anti-PD-1 immunotherapy of melanoma, YTHDF-2 has a promoting effect and FTO and alkbh5 have inhibitory effect. YTHDF-1 suppress anti-PD-L1 immunotherapy for colon cancer. FTO promotes PD-L1 expression in colon cancer. WTAP suppress tumor immunity of gastric cancer. RNA methylation regulates immunotherapy for lung cancer. FTO inhibits immunotherapy of acute myeloid leukemia. IGF2BP1, IGF2BP2, and METTL3 regulate immune microenvironment of nasopharyngeal carcinoma. YTHDC-2 has a promoting effect in tumor immunity of HNSCC. M^6^A methylation regulates tumor immunity of breast cancer. METTL3, KIAA1429, METTL14, YTHDF1, ALKBH5 regulate immune microenvironment of pancreatic adenocarcinoma.

RNA methylation plays an essential role in tumorigenesis and development. Abnormal RNA methylation has been confirmed to be associated with human cancer (104, 105). In addition to directly affecting the occurrence and development of tumors, RNA methylation affects tumors by regulating tumor immunity. The immune system plays an important role in the development, growth, invasion and metastasis of tumors. On the one hand, immune cells attack tumor cells and inhibit the development of tumors; on the other hand, immune escape by tumors promotes their development, growth, invasion and metastasis (106). RNA methylation affects tumors through the regulation of related components in the immune system (Table 1 and Figure 3).

**Table 1:** RNA methylation regulates tumor immunity in various cancers.

**Cancer type**	**Regulator**	**Function**	**References**
Melanoma	YTHDF-1	Inhibits tumor immunity	(58)
	YTHDF-2	Enhance anti-PD-1 immunotherapy	(107)
	ALKBH5 and FTO	Inhibits anti-PD-1 immunotherapy	(108)
Colon carcinoma	YTHDF-1	Suppress anti-PD-L1 immunotherapy	(58)
	FTO	Promotes PD-L1 expression	(109)
	METTL3 and METTL14	Suppress anti-PD-1 response	(110)
Gastric cancer	WTAP	Suppress tumor immunity	(111)
	m6A regulators	Correlation with immune checkpoints	(112, 113)
Lung cancer	m6A regulators	Correlation with immunotherapy response	(114)
Acute myeloid leukemia	FTO	Inhibits immunotherapy	(115)
Nasopharyngeal carcinoma	m6A regulators	Correlation with infiltration of immune cells	(116)
Breast cancer	m6A regulators	Correlation with anti-tumor immunity	(117)
	METTL14 and ZC3H13	Promotes tumor immunity	(118)
Pancreatic adenocarcinoma	m6A regulators	Correlation with anti-tumor immunity	(119)
HNSCC	YTHDC-2	Promotes anti-tumor immunity	(120)

### Melanoma

Tumor neoantigens play an important role in tumor immunotherapy (121). M^6^A methylation regulates the long-lasting specific immunity of tumor neoantigens through m^6^A-binding proteins. In a melanoma model in mice, YTHDF1-deficient mice showed slower tumor growth and longer survival than wild-type mice. By restricting the lysosomal protease expression of DCs cross-presentation is enhanced so that CD8^+^ T cells can better exert antitumour immune effects (58).

M^6^A modification inhibits the growth, metastasis, invasion and viability of melanoma cells by regulating tumor immunity. As a demethylase, FTO has been shown to promote the development of melanoma. PD-1 mRNA, as a target of FTO, is demethylated. M^6^A-methylated PD-1 mRNA is recognized by YTHDF2, which promotes its degradation. Therefore, the knockout of FTO decreases the level of PD-1 mRNA, which means lower expression of PD-1. In a melanoma mouse model treated with anti-PD-1 immunotherapy, knockout of FTO significantly inhibited tumor growth (107). Knockout of YTHDF-1 or blocking methylation of lysosomal protein RNA in DC inhibits tumor immune escape in melanoma patients. This provides a method of immunotherapy. The overexpression of YTHDF-2 in immune cells or the enhancement of PD-1 RNA methylation reduces the expression of PD-1 in immune cells, which enhances the effect of anti-PD-1 immunotherapy. These provide help in the clinical treatment of melanoma.

A recent study found that in anti-PD-1 therapy for melanoma, the deletion of ALKBH5 decreased the levels of infiltrating Tregs and polymorphonuclear myeloid derived suppressor cells (PMN-MDSCs), while the level of dendritic cells increased. In the process of this regulation, the m^6^A modification of the target gene Mct4/Slc16a3 increased, which means a higher stability and level. Mct4 is the key enzyme that catalyzes the rapid transport of lactic acid across the plasma membrane. And extracellular lactic acid directly affects the recruitment of Tregs and MDSC in the tumor microenvironment. A small-molecule ALKBH5 inhibitor, which can enhance the effect of anti-PD-1 therapy for melanoma, was found in this study (108).

### Colon Carcinoma

Similarly, immunoregulation of RNA methylation is essential in colon cancer therapy. In a colon cancer tumor-bearing mouse model, YTHDF1-deficient mice show tumor growth inhibition and longer survival than control mice. Early activation of T cells in YTHDF1-deficient mice was enhanced, and the immune infiltration levels of CD8^+^ cytotoxic T cells and natural killer cells were higher, while the infiltration of bone marrow-derived suppressor cells was lower. DCs showed a stronger ability to cross-present tumor antigens in this process. YTHDF1-deficient mice showed a higher cure rate with anti-PD-L1 treatment than control mice (58). Similar to the melanoma mentioned above, limiting the expression of YTHDF-1 is also used in the treatment of colon cancer and shows a higher survival rate in anti-PD-L1 immunotherapy for colon cancer. These results provide a new idea for immunotherapy of colon cancer. In addition, the detection of YTHDF-1 is used to evaluate the prognosis of patients with colon cancer.

As a demethylase, FTO is thought to regulate the methylation of PD-L1mRNA to affect the expression of PD-L1 in colon cancer cells. The detailed regulation mechanism of this process is not clear, but with the further research, it will provide new ideas for immunotherapy of colon cancer (109). In addition, in mismatch-repair-proficient or microsatellite instability-low (pMMR-MSI-L) colorectal cancer and melanoma, the deletion of METTL3 and METTL14 increased the infiltration of CD8+T cells and the secretion of IFN- γ, Cxcl9 and Cxcl10, and enhanced the anti-PD-1 response. The absence of METTL3 and METTL14 reduced the m6A modification of STAT1 and IRF1 mRNA and the YTHDF2-mediated mRNA degradation to increase the levels of STAT1 and IRF1 which participate in IFN- γ-Stat1-Irf1 signal axis and play an important role in anti-PD-1 response (110).

### Gastric Cancer

WTAP is an important component of the RNA methylase complex and recruits m^6^A methyltransferases. WTAP is present at a high level in the tumor tissues of patients with gastric cancer and is significantly related to the prognosis of patients. A study found that patients with high expression of WTAP had lower levels of Tregs and CD4 memory-activated T cells. High infiltration of Tregs and CD4 memory-activated T cells can improve the prognosis of patients with gastric cancer. This means that high expression of WTAP affects the prognosis of patients with gastric cancer by suppressing tumor immunity. Therefore, detecting the level of WTAP could evaluate the prognosis of patients with gastric cancer and provide a direction for immunotherapy of patients with gastric cancer (111).

By analyzing 1,938 cases of gastric cancer, Zhang et al. concluded that m^6^A modification is significantly related to tumor immune microenvironment and tumor immunotherapy. A recent study by Mo et al. build an m^6^A risk score: FTO × 0.127+YTHDF1 × 0.004+KIAA1429 × 0.044+YTHDC2 × 0.112-RBM15 × 0.135-ALKBH5 × 0.019-YTHDF2 × 0.028. It is considered that the risk score can be used as an independent prognostic index of gastric adenocarcinoma. Low risk score is associated with high expression of immune checkpoints, such as PD-1, PD-L1, and CTLA-4, which means that the risk factor can be used to evaluate the efficacy of immunotherapy for gastric cancer. The above view has not been clinically verified, but it suggests that the methylation of RNA can be used as an independent marker for predicting prognosis and guiding immunotherapy in patients with gastric cancer. With further understanding of the relationship between RNA methylation and immunity, altering methylation modification may provide help for personalized and accurate treatment of gastric cancer (112, 113).

### Lung Cancer

Immunotherapy plays an important role in the treatment of lung cancer by affecting RNA methylation. In a study on the prognosis of lung cancer, it was found that high-risk patients who responded better to immunotherapy had more methyltransferases than high-risk patients who did not respond well to immunotherapy. This may suggest that RNA methylation has the capacity to predict prognosis and guide the use of drugs in immunotherapy for lung cancer (114).

### Acute Myeloid Leukemia

A recent study has found that some FTO inhibitors have been identified to play a role in tumor immunotherapy by interfering with RNA methylation. In addition to the common immune checkpoints such as PD-L1, LILRB4 also plays an important role in immunotherapy in AML. By inhibiting the methylation of LILRB4 regulated by FTO, it can affect the expression of LILRB4, make AML cells more sensitive to T cell toxicity, and play a synergistic role in the immunotherapy of AML. Previous studies have found FTO inhibitors suitable for clinical use, which will be of great help to the immunotherapy of AML (115).

### Nasopharyngeal Carcinoma

In a recently study, Lu build a risk model of nasopharyngeal carcinoma with m^6^A-related genes, the risk score = IGF2BP1 × 0.161557 + IGF2BP2 × 0.01313 + METTL3 × (−0.0624), which is considered an independent prognostic factor. In nasopharyngeal carcinoma patients, the infiltration of immune cells (B naïve cells, B memory cells, CD8T, CD4 memory T activated, T gamma delta, Mast cells resting, and activated Mast cells) is significantly increased and the infiltration of immune cells (CD4 memory T resting, Macrophages M0, and Macrophages M2) is significantly decreased in the high risk score group compared to low risk score group. This finding revealed the potential role of m^6^A modification in nasopharyngeal carcinoma, providing novel insight into nasopharyngeal carcinoma prognosis and therapeutic strategies (116).

### Breast Cancer

A recent study analyzed the RNA sequence data of 24 m6A regulators in 775 breast cancer patients in TCGA. Patients with performing consensus clustering of 24 commonly reported m6A regulators based on the RNA expression data from TCGA, were divided into two groups. The two groups were significantly correlated with the number of tumor infiltrating CD8+T cells, helper T cells, activated NK cells, macrophage M2 and regulatory T cells. It was also significantly correlated with the expression of PD-L1, TIM3, LAG3, and CCR4, which are well-known T-cell exhaustion markers and important targets in immunotherapy. Compared with the low methylation group, the number of tumor infiltrating CD8+T cells, helper T cells and activated NK cells increased significantly, while the expression of PD-L1, PD-L2, TIM3, and CCR4 decreased significantly in the high methylation group. These results suggest that m6A regulators are closely related to the anti-tumor immunity of breast cancer. However, these analyses come from bioinformatics tools, and there are no further experiments to verify them. However, this provides comprehensive evidence for the further study of m6A regulators in breast cancer, and provides new clues for the epigenetic regulation of anti-tumor immune response (117).

Another study based on bioinformatics suggests that the low expression of METTL14 and ZC3H13 mRNA indicates a poor prognosis of breast cancer. The expression of METTL14 and ZC3H13 in breast cancer was positively correlated with the infiltration of CD4+T cells, CD8+T cells, neutrophils, macrophages and DC, and negatively correlated with Treg cells. The study puts forward a scientific hypothesis: the low expression of METTL14 and ZC3H13 leads to the decrease of Adenomatous polyposis coli (APC) mRNA methylation, which in turn reduces the stability of APC mRNA. The decrease of APC level leads to the abnormal activation of Wnt signal pathway which plays an important role in the regulation of tumor immune microenvironment and regulate the infiltration and activity of various types of T cells in tumor (118).

### Pancreatic Adenocarcinoma

Based on the analysis of the expression of m6A methylation regulatory gene in pancreatic Adenocarcinoma and normal tissue data from TCGA and GEO database, a recent study suggested that m6A methylation regulatory gene is related to the prognosis and immune microenvironment of pancreatic Adenocarcinoma, and constructed a m6A-related risk signature: risk score= −0.087 × METTL3 + 0.233 × KIAA1429-0.132 × METTL14-0.035 × YTHDF1-0.0286 × ALKBH5. In the high risk group, the infiltration of macrophage M0 and macrophage M2 increased, while the levels of B cells, CD8+T cells and regulatory T cells decreased. These results suggest that m6A regulatory gene regulates the immune microenvironment of pancreatic Adenocarcinoma through a comprehensive and complex process. Similarly, these results have not been verified by experiments, and the specific regulation mechanism has not been clarified (119).

### Head and Neck Squamous Cell Carcinoma

YTHDC2 is a reader of N6-methyladenosine. Recent studies have found that YTHDC2 is associated with the level of immune infiltration of B cells, CD8+T cells, CD4+T cells, neutrophils and dendritic cells in head and neck squamous cell carcinoma (HNSCC). Through the analysis of gene expression and prognosis of HNSCC patients in GEO, Oncolnc, Kaplan-Meier plotter, TIMER and other databases, it was found that YTHDC2 could be an independent tumor suppressor gene to judge the prognosis of HNSCC, and the expression level of YTHDC2 was positively correlated with the infiltration level of CD4+T cell subsets. However, the correlation between YTHDC2 and CD4+T cell subsets and its specific mechanism need further experimental study (120).

## Dual Roles of RNA Methylation in Immunity

As mentioned above, RNA methylation has dual effects on tumor regulation of immunity. In different types of tumors, RNA methylation can have a promoting or inhibitory role. The existing data suggest that RNA methylation also has dual roles in immunity.

The absence of m^6^A inhibited the differentiation and proliferation of T cells, which could prevent colitis in experimental mice. However, in Tregs, the absence of m^6^A inhibited their inhibitory function, which could lead to autoimmune diseases in experimental mice (21, 85).

On the one hand, methylation promotes immunity, while demethylation suppresses immunity. After methylation, CD40, CD80, and TIRAP on DCs promote the activation of DCs and enhance immunity. During the migration of DCs stimulated by CCR7, lnc-Dpf3 methylation promotes the migration and maturation of DCs, leading to abnormal inflammation. In melanoma cells, the inhibition of demethylation enhances tumor immunity. After the methylation of antiviral transcripts in cells is erased by demethylases, innate immunity is suppressed (34, 81, 107). On the other hand, methylation weakens the antigenicity of RNA and suppresses the immune response. After methylation modification, the immunogenicity of RNA is weakened or disappears, and innate immunity is no longer triggered. The methylation of mRNAs encoding lysosomal proteases inhibits the presentation of tumor antigens, resulting in tumor immune escape. After methylation of type I interferon mRNA, mRNA degradation increased, interferon production decreased, and innate immunity was suppressed (58, 102).

## Clinical Implications and Future Directions

The immune system is the host defense system against infection and disease. As a kind of genetic post-modification, RNA methylation not only participates in the immune response, but also participates in the occurrence and development of tumor by affecting splicing, nucleation, stability and immunogenicity of RNA. In addition to eliminating the immunogenicity of therapeutic RNA and treating immune diseases, RNA methylation provides a broader prospect for tumor immunotherapy.

In recent years, emerging tumor immunotherapy strategies have brought new hope to tumor patients. Some immunotherapeutic drugs, such as PD-1/PD-L1 inhibitors, have been approved for the clinical treatment of melanoma, lung cancer and colorectal cancer (122, 123). In melanoma, the deletion of YTHDF-1 enhances the anti-tumor immune response. Knock out FTO or ALKBH-5 enhances anti-PD-1 immunotherapy. In colon cancer, knockout YTHDF-1 enhances PD-L1 immunotherapy. The deletion of METTL3 and METTL14 enhances the anti-PD-1 response. In gastric cancer, high expression of WTAP suppresses tumor immunity and affects prognosis. In AML, suppression of FTO expression enhances immunotherapy. By targeting these methylation regulatory molecules, it can directly enhance tumor immunotherapy. In nasopharyngeal carcinoma, breast cancer, Pancreatic adenocarcinoma and HNSCC, using bioinformatics tool to analyze the database, it is found that many modulators have a strong correlation with tumor immunity and immunotherapy. Most of these conclusions have not been confirmed by experiments, but they provide a lot of directions for tumor immunotherapy. For example, these modulators can be used as biomarkers for biological prediction in immunotherapy and as targets to improve the response to immunotherapy. In addition, it can also be used alone or in combination with other drugs to treat tumor. For example, as a broad-spectrum antiviral drug, Ribavirin has been found in recent years to be used as an antitumour drug to participate in the treatment of a variety of tumors. It can not only be effective as a monotherapy, but also has been shown to enhance the effect of radiotherapy or chemotherapy (124). Previous study has found that Ribavirin competes with eIF4E to combine with m^7^G to block the translation of mRNA and play a role in the treatment of tumors. With the progress of RNA methylation research, the combination of Ribavirin and drugs regulating m^7^G in the treatment of tumor is also worth looking forward to (125).

Compared with RNA methylation, DNA methylation has become the focus of cancer research earlier. Some DNA methylation drugs have been widely used clinically, such as Azacytidine and Decitabine. Previous studies have found that the DNA demethylation drug Azacytidine inhibits the RNA transferase DNMT2 to regulate tRNA methylation (126). In a recent study, RNA methylation modification affected resistance in leukemia treated with the DNA demethylation drug 5-Aza (127). In the future, the combined application of DNA demethylation drugs and RNA methylation drugs may provide new treatments for tumors. In addition, RNA methylation modulators can also be used as new markers for predicting the sensitivity of DNA methylation drugs. Those DNA methylation drugs that can regulate RNA methylation also have the potential to be applied to RNA methylation therapy or immunotherapy.

## Conclusion

RNA methylation, which regulates not only mRNAs but also ncRNAs, has been proven to be able to regulate a variety of biological functions, especially tumor immunity. RNA methylation can regulate a variety of immune cells and has a variety of regulatory modes and mechanisms, such as regulating the differentiation of T cells, the state of Treg cells, and the maturation of DCs. In addition, modifications can also regulate a variety of immune cytokines, such as IL-17. RNA methylation regulates human immune status and tumor immunity. RNA methylation also shows dual roles in regulating immunity, which affects immune function. In a variety of tumors, RNA methylation affects the effectiveness of tumor immunotherapy by regulating immune function. At present, research on the relationship between RNA methylation and tumor immunity is still in its infancy, and more in-depth research is needed to explore the mechanism. RNA methylation has the potential to provide new ideas for the identification of tumor patients who will be sensitive to a given drug, the determination of prognostic indicators, and research and development of targeted drugs.

## Author Contributions

ZS and JS provided direction and guidance throughout the preparation of this manuscript. MZ wrote and edited the manuscript. WY reviewed and made significant revisions to the manuscript. WZ collected and prepared the related papers. All authors read and approved the final manuscript.

## Conflict of Interest

The authors declare that the research was conducted in the absence of any commercial or financial relationships that could be construed as a potential conflict of interest.

## Abbreviations

ALKBH5: alkylation repair homing protein 5
CCR7: chemokine-C receptor 7
DCs: Dendritic cells
DDX46: DEAD-box 46
DNMT: DNA methyltransferase
ERK: extracellular regulated protein kinases
FTO: fat mass and obesity-associated protein
HIF-1α: hypoxia inducible factor-1α
IGF2BP1/2/3: insulin-like growth factor 2 mRNA binding protein 1/2/3
IL-17: interleukin 17
IL-2: interleukin 2
IL-7: interleukin 7
IRF3: Interferon regulatory Factor 3
LDHA: lactate dehydrogenase A
Mavs: mitochondrial antiviral signaling protein
METTL1: methyltransferase-like 1
METTL14: methyltransferase-like 14
METTL16: methyltransferase-like 16
METTL3: methyltransferase-like 3
MSI: microsatellite instability
m^6^A: N6-methyladenosine
m^5^C: 5-methylcytosine
m^7^G: N7-methylguanosine
ncRNAs: non-coding RNAs
Nsun2: NOP2/Sun RNA methyltransferase 2
PD-L1: Programmed cell death 1 ligand 1
PD-1: programmed cell death protein 1
PKR: protein kinase R
RBM15/15B: RNA binding motif protein 15/15B
RIG-I: retinoic acid-inducible gene I
snoRNA: small nucleolar RNA
snRNA: small nuclear RNA
SOCS: Suppressor of cytokine signaling
STAT5: Signal transducers and activators of transcription 5
TIRAP: Toll-interleukin1 receptor domain containing adaptor protein
TLRs: Toll-like receptors
TMB: tumor mutational burden
Traf3: TNF receptor associated factor 6
Traf6: TNF receptor associated factor 6
Tregs: Regulatory T cells
WDR4: WD repeat domain 4
WTAP: Wilms tumor 1-associated protein
YTHDC1/2: YTH domain-containing 1/2
YTHDF1/2/3: YTH N6-methyladenosine RNA binding protein 1/2/3
ZCCHC4: CCHC zinc finger-containing protein 4
ZC3H13: Zinc finger CCCH domaincontaining protein 13.
